# COVID-19-Induced Myocarditis and mRNA Vaccine-Related Pericarditis: A Case Report

**DOI:** 10.7759/cureus.28440

**Published:** 2022-08-26

**Authors:** Clara L Voltarelli, Luiza Silva, Mariana Longo, Stefany Ferraria, Lucas L Martins, Guilherme Nazar, Tiago Magalhães, Rafael Miyazima, Gustavo Lenci Marques

**Affiliations:** 1 Internal Medicine Department, Federal University of Parana, Curitiba, BRA; 2 Radiology Department, Federal University of Parana, Curitiba, BRA; 3 School of Medicine, Pontifical Catholic University of Paraná, Curitiba, BRA

**Keywords:** perimyocarditis, covid 19, bnt162b2 mrna vaccine, covid and myocarditis, pericardial diseases, heart failure

## Abstract

Acute inflammatory cardiac disease is an increasing cause of COVID-19 vaccine-induced complications. We report a case of acute pericarditis following the second dose of the COVID-19 vaccine (BNT162b2) in a 49-year-old woman with previous COVID-19-induced myocarditis and heart failure. A clinical presentation compatible with acute decompensated heart failure elevated troponin levels and a cardiac-MRI showing myocardial fibrosis and inflammatory pericardial effusion led to the diagnosis of perimyocarditis. She was treated with non-steroidal anti-inflammatory drugs (NSAIDs) and colchicine. Her condition improved in eight days. Physicians should be aware of the possible diagnosis of pericarditis and/or a myocardial injury after COVID-19 infection and vaccination.

## Introduction

Myocarditis can be defined as an exacerbated inflammatory response in the cardiac muscle. Typically, it affects more men than women and shows a higher incidence among children, adolescents and young adults [1,2]. Clinical presentation is highly variable and usually includes chest pain, dyspnea and palpitations, associated with troponin elevation and imaging findings on the echocardiogram (e.g., abnormal wall motion), electrocardiogram (e.g. ST segment or T-wave abnormalities, ventricular and supraventricular arrhythmias, intraventricular conduction delay) or magnetic resonance imaging (e.g., myocardial edema, non-ischemic myocardial injury pattern, pericarditis, left ventricular systolic dysfunction) [2].

Viruses are known agents associated with this condition, as infections can cause an imbalanced immune response, culminating in excessive inflammation and cardiomyocyte damage. Such inflammatory cascade could be triggered by the stimulation of innate immunity, which activates signaling pathways, including those related to tyrosine kinases p56, Fyn and Abl, and toll-like receptors (TLRs), particularly TLR-3 and TLR-4. Simultaneously, T-killer cells, and T and B lymphocyte activation can cause subacute and chronic inflammation, leading to necrosis, fibrosis and myocardial remodeling [3]. In addition, a cytokine storm involving the activation of intracellular inflammatory and fibrogenic pathways also seems to contribute to this process [4]. 

Acute perimyocarditis (AP) and myocardial injury has been reported as potentially severe complication after the COVID-19 vaccine [5-8] , especially after its second dose, which causes a significantly stronger immune response when compared to its first dose [9]. Cases of myocardial injury following RNA vaccines have been reported, especially in adolescents and adults under 40 years [10]. Although potentially severe, most cases reported progressed with mild or moderate severity [11].

Myocarditis is commonly underdiagnosed, leading to the hypothesis that vaccine-related myocarditis is under-represented in the medical literature [12]. The mechanism of this condition most likely involves immune-mediated pathways leading to myocyte death due to the expression of spike protein on the surface of cardiomyocytes [10].

## Case presentation

A 49-year-old black female presented to the hospital 12 days after receiving the second dose of the Pfizer-BioNTech COVID-19 vaccine (BNT162b2; Pfizer, Inc; Philadelphia, PA) vaccine. Her medical history included type 2 diabetes and hypertension, for which she took losartan 50 mg twice a day and metformin 500 mg twice a day. Two months earlier, the patient was diagnosed with myocarditis and pulmonary embolism due to a COVID-19 complication, which led to heart failure with reduced ejection fraction, and for which she took rivaroxaban 20 mg daily, spironolactone 25 mg daily, carvedilol 12.5 mg twice a day and furosemide 40 mg once a day.

**Figure 1:**
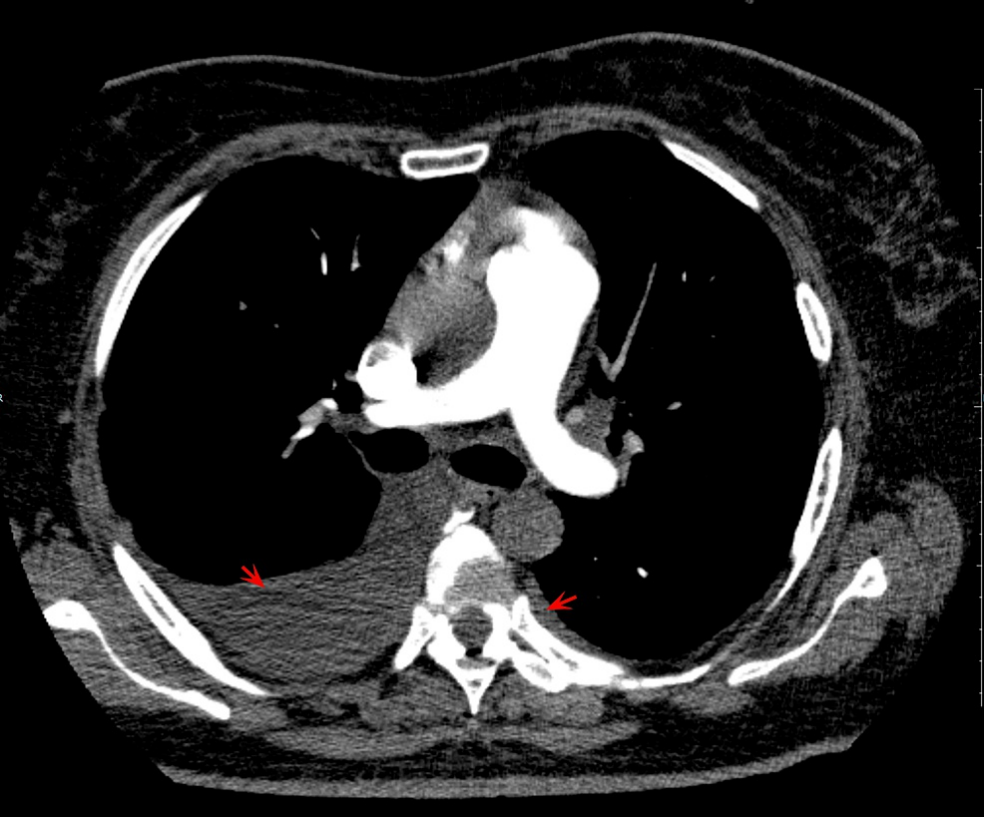
CT pulmonary angiogram showing bilateral pleural effusion (arrows), larger in the right hemithorax.

**Figure 2:**
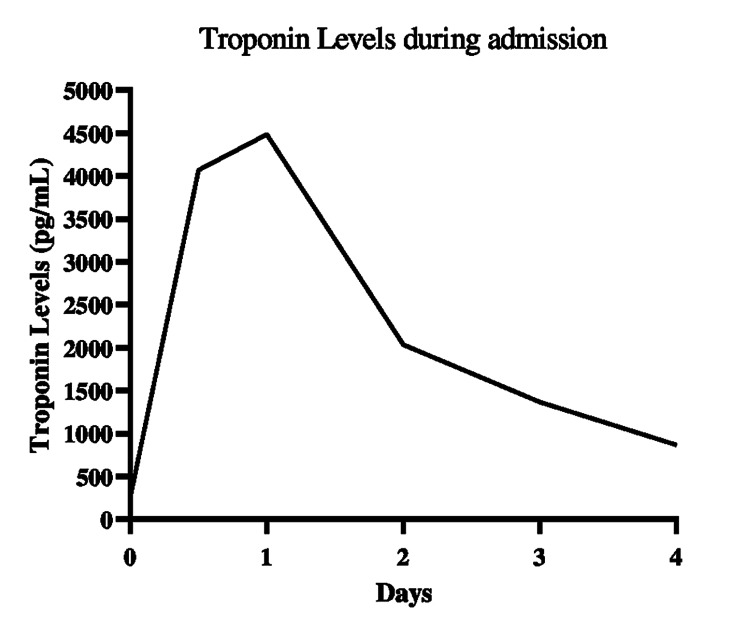
Graph showing troponin levels during the patient’s admission Patient's troponin levels reached peak at 4486.2 pg/mL on the second day's admission. At discharge, troponin was 870 pg/mL. Reference range is < 15.6 pg/mL.

Upon arrival, the patient presented chest pain, which worsened with breathing, nausea and vomiting. The vital signs included a heart rate of 90 beats per minute and blood pressure of 100/80 mmHg. Pulmonary sounds were absent on the right lung base and there were signs of poor perfusions, such as capillary refill time (CRT) longer than 5 seconds. The initial electrocardiogram showed sinus rhythm, left bundle branch block and left axis deviation. A computed tomography (CT) angiography (Figure 1) showed pleural effusion, larger in the right hemithorax. The significant lab results are summarized in Table 1 and Figure 2.

**Table 1:** Lab results performed during patient’s admission with normal reference range. C-reactive protein (CRP) levels slightly increased during the patient's admission. On the first day, CRP level was 0.45 mg/dL, increasing up to 0.53 on the fourth day's admission.

	Result	Reference Range
Creatinine	0.81 mg/dL	0.57 to 1.11 mg/dL
C-Reactive Protein	0.53 mg/dL	< 0.5 mg/dL
Procalcitonin	0.09 ng/mL	< 0.5 ng/mL
pH	7.5	7.31 to 7.41
PCO2	22.8 mmHg	41 to 51 mmHg
Lactate	2.84 mmol/L	0.36 to 1.39 mmol/L
PO2	95.1 mmHg	30 to 40 mmHg
HCO3	14.7 mmol/L	23 to 29 mmol/L

Based on the patient’s medical record, this clinical presentation was interpreted as acute decompensated heart failure stage C (“cold and wet"). The patient developed low blood pressure, which required transfer to the ICU. Due to the hypothesis of cardiogenic shock, the patient was treated with dobutamine infusion and her condition stabilized.

**Figure 3:**
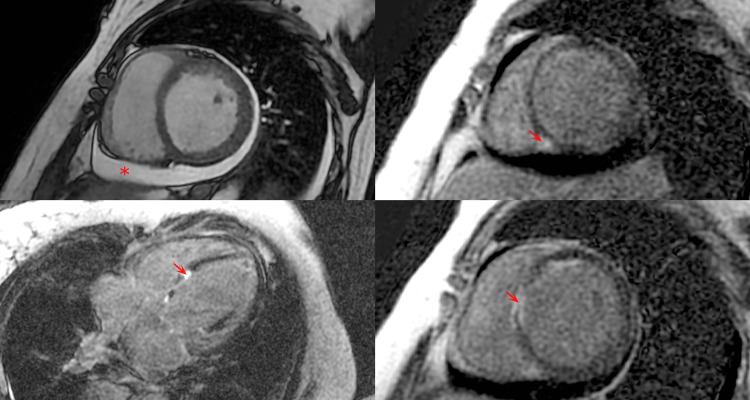
Cardiac MRI performed during the patient’s admission showing areas of late gadolinium enhancement (fibrosis) in the basal septal and medial inferoseptal walls (arrows) in a non-vascular regional distribution pattern and pericardial effusion (asterisk).

On the third day of admission, a transthoracic echocardiogram showed left ventricular eccentric hypertrophy, systolic dysfunction due to global hypokinesia, pulmonary hypertension, pericardial effusion and left ventricle ejection fraction of 33%. On the eighth day of admission, the patient was stable on the hospital ward and no longer receiving dobutamine, when a new transthoracic echocardiogram showed a left ventricle ejection fraction of 19%. The significant decrease in systolic function suggested a new episode of myocarditis. A cardiac MRI showed mesocardial fibrosis in a non-vascular regional distribution pattern and inflammatory pericardial effusion (Figure 3), despite the absence of T2-weighted myocardial edema, leading to the diagnosis of perimyocarditis. The patient was treated with naproxen 500mg twice a day and colchicine 0.5 mg twice a day and showed significant clinical improvement.

On her previous admission, due to COVID-19 infection, an echocardiogram already showed left ventricular eccentric hypertrophy, systolic dysfunction due to global hypokinesia, and pulmonary hypertension. The left ventricle ejection fraction was 25%.

## Discussion

Vaccine-associated cardiac inflammatory disease is defined as any sort of cardiac inflammatory syndrome occurring shortly after vaccination without another identifiable cause. Our patient developed symptoms 12 days after the second dose of the mRNA vaccine, which shows a temporal association between mRNA COVID-19 vaccination and AP.

Lazaros et al. reported nine cases of post-vaccine AP, of which six (approximately 66%) were caused by Pfizer’s BNT162b2 vaccine. This study also shows that young adults are more affected, specifically ages between 18 and 29 years, as well as women over men [8].

Other studies show that the systemic inflammatory response is significantly higher after the second vaccine dose [9], which can explain why our patient did not show similar symptoms after the first dose. Such an immune reaction, although substantial, is not greater than the cytokine storm caused by the actual COVID-19 disease [4,7]. This could explain why the reported clinical presentation was mild when compared to the same patient’s previous hospitalization, which led to cardiac failure as a sequel.

Due to such a medical history of post-COVID-19 myocarditis, our patient might have had an increased risk of recurrence after the COVID-19 vaccine. Considering this background, acute decompensation due to ischemia or infections had to be, necessarily, ruled out. Acute pulmonary embolism was also excluded through CT angiography. There was no evidence of an active viral illness or autoimmune disease. The treatment included colchicine and Non-steroidal anti-inflammatory drugs (NSAIDs), although there is no standard treatment for vaccine-induced perimyocarditis to this date.

Reports on worldwide medical literature show that nearly 2.3% of patients infected by COVID-19 presented clinical or subclinical myocarditis, raising the hypothesis that this complication is no longer anecdotal [6]. Although rare, post-vaccine cases of myocarditis are also reported, reaching an incidence of 2.13 in the 100,000 population [11]. The incidence of post-vaccine AP in Europe and the United State combined reached 670 cases in more than 392 million doses of the BNT162b2 vaccine [8]. In Brazil, from the beginning of the mRNA vaccine administration until March of 2022, 0.05 reports of pericarditis and/or myocarditis were registered for every 100,000 doses [13]. Such rates are inferior when compared to global data, probably due to under-reporting.

Because myocarditis due to COVID-19 infection is caused by an intense immune response [4], it is inferred that it is possible to present perimyocarditis due to the COVID-19 vaccine. Because the immune response stimulated through vaccination is controlled, it is speculated that the clinical presentation of cardiac inflammatory diseases in that background is mild to moderate.

The benefits of mRNA vaccines (prevention of COVID-19 disease and associated hospitalization) seem to outweigh the risk of myocarditis related to vaccination, although the net benefit varies among different ages and sex [2]. In this scenario, previous myocarditis is not an absolute contraindication to vaccination, but clinicians must assess individual risk factors before counseling further doses [14,15]. The case of our patient is an example of how serious adverse effects can present in this context.

## Conclusions

As vaccination coverage increases, patients presenting with chest pain, shortness of breath and elevated cardiac markers should be differentially assessed for inflammatory cardiac diseases. In this context, the medical history should include prior COVID-19 vaccination and infection, in order to establish an early diagnosis and proper management.

There is little data about the safety of vaccination in patients with previous myocarditis induced by COVID-19. Thus, further studies are necessary to determine whether the prevalence and severity of this event are higher than among individuals of similar age in the general population and to assess the long-term consequences of inflammatory cardiac diseases related to COVID-19 disease or vaccines. Hence, cases of myocarditis and pericarditis after COVID-19 vaccination should be thoroughly reported to the respective health departments, as the prevalence of these events is crucial to stimulate early diagnosis in order to predict and avoid poor outcomes.
